# An infection and pathogenesis mouse model of SARS‐CoV‐2‐related pangolin coronavirus GX_P2V(short_3UTR)

**DOI:** 10.1002/mlf2.12122

**Published:** 2026-03-30

**Authors:** Lai Wei, Shuiqing Liu, Shanshan Lu, Shengdong Luo, Xiaoping An, Huahao Fan, Erguang Li, Lihua Song

**Affiliations:** ^1^ College of Life Science and Technology Beijing University of Chemical Technology Beijing China; ^2^ Research Center for Clinical Medicine The Fifth Medical Center of PLA General Hospital Beijing China; ^3^ State Key Laboratory of Pharmaceutical Biotechnology, Medical School Nanjing University Nanjing China

## Abstract

SARS‐CoV‐2‐related pangolin coronavirus GX_P2V(short_3UTR) is highly attenuated, but can cause mortality in a specifically designed human *ACE2*‐transgenic mouse model, making it a surrogate model for evaluating the efficacy of vaccines and drugs against SARS‐CoV‐2.

Two SARS‐CoV‐2‐related pangolin coronaviruses, GD/2019 and GX/2017, were identified before the COVID‐19 outbreak^1^, ^2^. The respective isolates, termed pCoV‐GD01 and GX_P2V, were cultured in 2020 and 2017, respectively^2^, ^3^. The infectivity and pathogenicity of these isolates have been studied^4^, ^5^, ^6^. The pCoV‐GD01 isolate, which has higher homology with SARS‐CoV‐2, can infect and cause disease in both golden hamsters and human *ACE2* (hACE2) transgenic mice^4^. In contrast, GX_P2V is highly attenuated in previously tested animals, such as golden hamsters, BALB/c mice, and two types of hACE2 transgenic mice^5^, ^6^. We previously reported that the early passaged GX_P2V isolate was actually a cell culture‐adapted mutant named GX_P2V(short_3UTR), which possessed a 104‐nucleotide deletion at the 3′‐UTR^6^. In this study, we analyzed its adaptive mutation in cell culture and assessed its pathogenicity in a unique CAG‐hACE2 transgenic mouse model. We found that GX_P2V(short_3UTR) can infect hACE2 transgenic mice, with high viral loads detected in both lung and brain tissues, which are correlated with the strong expression of hACE2 in these tissues. This infection resulted in mortality in the hACE2 transgenic mice. We surmise that the cause of death may be linked to the occurrence of a late brain infection.

We first analyzed the adaptive mutations of the GX_P2V(short_3UTR) mutant in cell cultures through random cloning and sequencing. The passaged mutant was cloned through two successive plaque assays. Further, eight viral clones were chosen for next‐generation sequencing (National Genomics Data Center of China, GSA: CRA014225). When compared with the genome of the original mutant^6^, these clones shared four identical mutations: ORF1ab_D6889G, S_T730I, S_K807N, and E_A22D (Table S1). Then, Clone 7, named GX_P2V C7, was randomly selected for the evaluation of its viral pathogenicity in hACE2 transgenic mice (Figure 1A). The hACE2 transgenic mouse model expressing hACE2 under control of the CAG promoter was developed using random integration technology by Beijing SpePharm Biotechnology Company.

**Figure 1:**
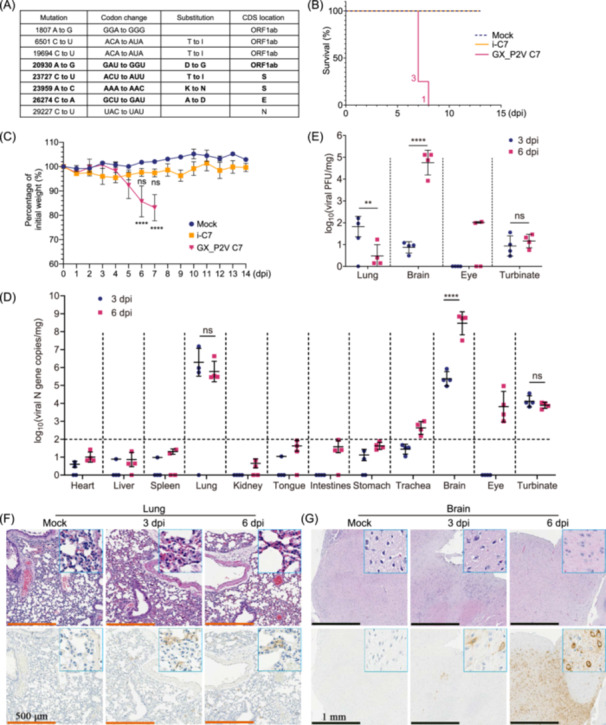
Characterization of a lethal infection model in human *ACE2* (hACE2) transgenic mice caused by attenuated SARS‐CoV‐2‐related pangolin coronavirus GX_P2V C7. (A) Mutations in GX_P2V C7 compared to the GX_P2V(short_3UTR) isolate (NCBI accession number: MW532698). The four conserved mutations in all eight clones of GX_P2V(short_3UTR) (Table S1) are shown in bold. (B) Survival of hACE2 transgenic mice following intranasal infection with GX_P2V C7 (*n* = 4), inactivated GX_P2V C7 (i‐C7; *n* = 4), and mock infection (*n* = 4). The number of deceased mice on each specific day is annotated on the left of the survival curve. (C) Percentage of the initial weight of hACE2 transgenic mice after intranasal infection with GX_P2V C7 (*n* = 4), i‐C7 (*n* = 4), and mock infection (*n* = 4). The statistical significance of the differences between mock‐infected (*n* = 4; blue dots) and GX_P2V C7‐infected (*n* = 4; red dots) or i‐C7‐infected mice (*n* = 4; orange dots) on 6 or 7 dpi is shown. The data represent the means ± SDs. (D) Quantification of GX_P2V N gene copies in the heart, liver, spleen, lung, kidney, tongue, intestines, stomach, trachea, brain, eye, and turbinate homogenates at 3 and 6 dpi (*n* = 4 per group). The limit of detection (LOD) for viral RNA loads in the original samples is shown to be 2.0 log_10_(copies/mg). The data represent the means of log_10_(copies/mg) ± SDs. The statistical significances of the comparisons in the lungs, brain, and turbinates are shown. (E) Infectious viral titers in lung, brain, eye, and turbinate homogenates were measured using a plaque‐forming assay at 3 and 6 dpi (*n* = 4 per group). The statistical significance of the differences in the lungs, brain, and turbinates is shown. The data represent the means of log_10_(PFU/mg) ± SDs. (F, G) Hematoxylin and eosin (H&E) staining (upper) and immunohistochemical (IHC) staining (lower) with an anti‐SARS‐CoV‐2 N‐specific antibody revealing viral antigen‐positive cells in the lung (F) and brain (G), as shown at high magnification in the inset. The scale bars were 500 μm (F) and 1 mm (G), respectively. **p < 0.01; ****p < 0.0001; ns, not significant. Two‐way analysis of variance, followed by Sidak's multiple comparison test. dpi, days postinfection.

Following this, we assessed whether GX_P2V C7 could cause disease in hACE2 transgenic mice by monitoring their daily weight and clinical symptoms. A total of four 6‐ to 8‐week‐old hACE2 transgenic mice were intranasally infected with a dosage of 5 × 10^5^ plaque‐forming units (PFUs) of the virus GX_P2V C7. Further, four mice were inoculated with inactivated virus, and four mock‐infected mice were used as controls. It is noteworthy that all the mice infected with the live virus succumbed to the infection within 7–8 days postinoculation, rendering a mortality rate of 100% (Figure 1B). The mice began to show a decrease in body weight starting from Day 5 postinfection, reaching a 10% decrease from their initial weight by Day 6 (Figure 1C). By Day 7 following infection, the mice displayed symptoms such as piloerection, a hunched posture, sluggish movements, and white eyes. Additionally, the criteria for the clinical scoring of the mice and the daily clinical scores postinfection with GX_P2V C7 are provided in Figure S1.

We then evaluated the tissue tropism of GX_P2V C7 in the hACE2 transgenic mice. Using the infection method described above, eight hACE2 transgenic mice were infected with live virus, eight mice were inoculated with inactivated virus, and eight mock‐infected mice were used as controls. The organs of four randomly selected mice in each group were dissected on Days 3 and 6 postinfection for the quantitative analysis of viral RNA and titer. We detected significant amounts of viral RNA in the brain, lungs, turbinates, eyes, and trachea of the GX_P2V C7‐infected mice (Figure 1D), whereas in other organs, such as the heart, liver, spleen, kidneys, tongue, stomach, and intestines, no or a low amount of viral RNA was detected. Specifically, in the lung samples, we detected high viral RNA loads on Days 3 and 6 postinfection, with no significant difference between these two‐time points (~6.3 as opposed to ~5.8 log_10_(copies/mg)). In the brain samples, on Day 3, we detected viral RNA in all four infected mice, with an average value of 5.4 log_10_(copies/mg). Notably, by Day 6, we observed exceptionally high viral RNA loads (~8.5 log_10_(copies/mg)) in the brain samples from all four infected mice (Figure 1D). On Days 3 and 6, the viral RNA loads in the turbinate samples were similar: approximately 4.1 and 3.9 log_10_(copies/mg), respectively. The viral RNA loads in the trachea and eyes of the mice surpassed the limit of detection only on Day 6, with values of 2.6 and 3.8 log_10_(copies/mg), respectively.

In addition, regarding the infectious viral titers, lung tissues on Day 3 postinfection had a value of ~1.8 log_10_(PFU/mg), which decreased to ~0.5 log_10_(PFU/mg) by Day 6. Importantly, the highest infectious titers were detected in the brain on Day 6, which was significantly greater than the highest infectious titers found on Day 3 (~0.9 as opposed to ~4.8 log_10_(PFU/mg); Figure 1E). Further, there were no significant differences in the infectious titers in the turbinate samples between Day 3 (~0.9 log_10_(PFU/mg)) and Day 6 (~1.2 log_10_(PFU/mg); Figure 1E). By Day 6, approximately 2.0 log_10_(PFU/mg) was detected in the eyes of two mice. Neither inactivated GX_P2V C7 nor mock infection caused death or any clinical symptoms in the mice (Figures 1B,C and S2).

In summary, in the mice infected with a live virus, the viral load in the lungs significantly decreased by Day 6, and both the viral RNA loads and viral titers in the brain samples were relatively low on Day 3 but substantially increased by Day 6. This finding suggests that severe brain infection during the later stages is the key cause of death in these mice.

To determine the mechanisms underlying GX_P2V C7‐induced death in hACE2 transgenic mice, we examined the pathological changes, the presence of viral antigens, and cytokine profiles in the lung and brain tissues of the mice on Days 3 and 6 postinfection (Figures 1F,G and S3 and S4). On both Days 3 and 6, compared to those of the control mice, the lungs of the infected mice showed no significant pathological alterations, with only minor inflammatory responses due to slight granulocyte infiltration (Figure 1F). On Day 3 postinfection, shrunken neurons were visible in the cerebral cortex of the mice. By Day 6, in addition to the shrunken neurons, there was focal lymphocytic infiltration around the blood vessels, although no conspicuous inflammatory reaction was observed (Figure 1G). Upon staining for viral nucleocapsid protein via immunohistochemistry, viral antigens were detected in both the lungs and brains on Days 3 and 6 postinfection, with extensive viral antigens notably present in the brain on Day 6 (Figure 1F,G).

These findings align with the viral RNA load assessments in the lung and brain tissues (Figure 1D). We also performed a Luminex cytokine assay to detect 31 cytokines–chemokines in the lung and brain tissues of the mice (Figures S3 and S4). Consistent with the pathological findings, slight increases or decreases were observed in the levels of many cytokines–chemokines in the lung and brain tissues compared to those in control tissues; however, the levels of key inflammatory factors, such as IFN‐γ, IL‐6, IL‐1β, and TNF‐α, did not change significantly.

In brief, these analyses revealed that GX_P2V C7 infection in hACE2 transgenic mice did not lead to severe inflammatory reactions—a finding that aligns with previous reports by Zhengli Shi's group using GX_P2V infection in two different hACE2 transgenic mouse models^5^, as well as with our previous finding of GX_P2V(short_3UTR) being highly attenuated in the golden hamster model^6^.

To the best of our knowledge, this is the first report to analyze the cell‐adapted mutations of pangolin coronavirus GX_P2V and to show that it can cause mortality in hACE2 transgenic mice. These findings are inconsistent with those of Zhengli Shi et al.,^5^ who tested the virulence of GX_P2V in two different hACE2 transgenic mouse models. It is highly likely that the high pathogenicity of GX_P2V C7 in our hACE2 transgenic mice is due to the strong expression of hACE2 in the mouse brain (Figure S5). In fact, under normal circumstances, both human and mouse brains show low expression of *ACE2* ^7^, ^8^. Furthermore, while Beijing SpePharm Biotechnology Company has not yet published a paper detailing the construction and characterization of this hACE2 transgenic mouse model, we were notified that these hACE2 transgenic mice had abnormal physiology, as indicated by their relatively reduced serum triglyceride, cholesterol, and lipase levels, compared to those of wild‐type C57BL/6J mice. The outcomes of the mouse infections in this study have no correlation with human infections and do not alter the fundamental nature previously shown by GX_P2V(short_3UTR) of being highly attenuated.

Currently, there is an urgent need for the development of broadly protective vaccines against pan‐SARS‐CoV‐2. However, the emergence of the next SARS‐CoV‐2 variant is unpredictable. The receptor‐binding domain of pangolin coronavirus GX_P2V(short_3UTR) shares 86.8% amino acid identity with SARS‐CoV‐2. Thus, this lethal infection model may be valuable in assessing the effectiveness of broad‐spectrum COVID‐19 vaccine candidates against unknown future variants. Moreover, this lethal infection model manifests productive viral infections; however, it has no obvious inflammatory responses in the main affected organs, the lungs and the brain, thereby providing a distinctive model to evaluate the efficacy of antiviral drugs in inhibiting viral replication *in vivo*. In summary, our study provides a unique perspective on the pathogenicity of GX_P2V and offers a model for assessing the efficacy of vaccines and drugs against SARS‐CoV‐2.

## ETHICS STATEMENT

All animals involved in this study were housed and cared for in an AAALAC (Association for Assessment and Accreditation of Laboratory Animal Care)‐accredited facilities. The procedure for animal experiments (IACUC‐2019‐0027) was approved by the Institutional Animal Care and Use Committee of the Fifth Medical Center, General Hospital of the Chinese People's Liberation Army, and complied with IACUC standards. This study was conducted in an ABSL‐3 biosafety laboratory and all operations were conducted in strict compliance with relevant biosafety management regulations.

## Supporting information

Supporting information.

## Data Availability

All the data supporting the findings of this study are available within the article and the Supporting Information, or from the corresponding author upon reasonable request.
